# Use of sera cell free DNA (cfDNA) and exovesicle-DNA for the molecular diagnosis of chronic Chagas disease

**DOI:** 10.1371/journal.pone.0282814

**Published:** 2023-09-08

**Authors:** Noelia Lozano, Mercedes Gomez Samblas, Eva Calabuig, María José Giménez Martí, Maria Dolores Gómez Ruiz, José Miguel Sahuquillo Arce, Sergio Sequera-Arquelladas, José Miguel Molina Moreno, M. Trelis, Antonio Osuna

**Affiliations:** 1 Area of Parasitology, Department of Pharmacy and Pharmaceutical Technology and Parasitology, University of Valencia, Valencia, Spain; 2 Servicio de Microbiología y Parasitología Clínica, Hospital Universitario y Politécnico La Fe-IIS La Fe, Valencia, Spain; 3 Grupo de Bioquímica y Parasitología Molecular (CTS 183), Departamento de Parasitología, Campus de Fuentenueva, Instituto de Biotecnología, Universidad de Granada, Granada, Spain; 4 Unidad de Enfermedades Infecciosas, Servicio de Medicina Interna, Hospital Universitario y Politécnico La Fe-IIS La Fe, Valencia, Spain; 5 Unidad de Enfermedades Infecciosas, Hospital Universitario Virgen de las Nieves, Granada, Spain; 6 Joint Research Unit on Endocrinology, Nutrition and Clinical Dietetics, University of Valencia-Health Research Institute La Fe, Valencia, Spain; Universidade Federal de Minas Gerais, BRAZIL

## Abstract

Chagas disease, a neglected tropical disease, is now considered a worldwide health concern as a result of migratory movements from Central and South America to other regions that were considered free of the disease, and where the epidemiological risk is limited to transplacental transmission or blood or organ donations from infected persons. Parasite detection in chronically ill patients is restricted to serological tests that only determine infection by previous infection and not the presence of the parasite, especially in patients undergoing treatment evaluation or in newborns. We have evaluated the use of nucleic acids from both circulating exovesicles and cell-free DNA (cfDNA) from 50 samples twice randomly selected from a total of 448 serum samples from immunologically diagnosed patients in whom the presence of the parasite was confirmed by nested PCR on amplicons resulting from amplification with kinetoplastid DNA-specific primers 121F-122R. Six samples were randomly selected to quantify the limit of detection by qPCR in serum exovesicles. When the nucleic acids thus purified were assayed as a template and amplified with kinetoplastid DNA and nuclear satellite DNA primers, a 100% positivity rate was obtained for all positive samples assayed with kDNA-specific primers and 96% when SAT primers were used. However, isolation of cfDNA for *Trypanosoma cruzi* and amplification with SAT also showed 100% positivity. The results demonstrate that serum exovesicles contain DNA of mitochondrial and nuclear origin, which can be considered a mixed population of exovesicles of parasitic origin. The results obtained with serum samples prove that both cfDNA and Exovesicle DNA can be used to confirm parasitaemia in chronically ill patients or in samples where it is necessary to demonstrate the active presence of the parasite. The results confirm for the first time the existence of exovesicles of mitochondrial origin of the parasite in the serum of those affected by Chagas disease.

## Introduction

*Trypanosoma cruzi* is a protozoan parasite etiologically responsible for American trypanosomiasis or Chagas disease (CD), a zoonotic disease among the so-called neglected tropical diseases. In addition to humans, the parasitism affects other mammals that can serve as reservoirs for the parasite. The insects involved in vector transmission are blood-sucking reduviids of the family Triatominae, with mammalian infection occurring as a result of contamination of the skin and mucous membranes with insect feces containing infective forms of the protozoan. Non-vectorial human infection can occur orally by ingestion of fruit juice contaminated with crushed insects or feces, by transfusion [1, 2] or by vertical transmission from mother to fetus [3]. These last two routes are epidemiologically important in areas where there is no vector transmission exists.

Chagas disease is endemic in a total of 21 countries in the Americas, from the southern United States to Argentina and Chile [4, 5], with approximately 10 million people infected and an annual incidence of 50,000 to 200,000 infections. For many years, the disease was considered confined to Mesoamerica and South America, but in recent decades it has spread to other parts of the world through migratory processes. Cases have been reported in the United States, Canada, European countries, Australia or Japan, areas with varying degrees of migration from endemic areas [6], making CD a public health problem in European countries such as Spain, Italy or Sweden, among others, where strict control measures have been adopted by blood banks [7], gynecological services [8] and transplant surgeries [9–11].

In the course of Chagas disease, there is an acute phase (ACD) that occurs in the first eight weeks immediately after infection. It is usually associated with a high number of circulating parasites in the blood, which facilitates both parasitological and molecular diagnosis, especially when patients present with pathognomonic symptoms, although the likelihood of this occurring is low. For this reason, most patients are referred to as being chronically ill without a diagnosis. The chronic phase (CCD), which develops 3 to 8 weeks after infection, may persist for decades either asymptomatically or with mild symptoms and very low levels of parasitemia. It is estimated that only 30% of cases develop disease-specific symptoms throughout the disease [12–14]. These symptoms include cardiac and/or gastrointestinal disturbances [15]. CCD poses the greatest epidemiologic risk of disease transmission in countries where infection by insect vectors does not occur, either as a consequence of blood transfusion or transplantation, or as a consequence of transplacental transmission from mother to fetus. Due to the low parasitemia in blood, detection of the parasite is very difficult and new techniques and/or molecular markers are being developed to demonstrate the presence of the parasites or secreted products that indicate their active presence [16–20].

Diagnostic methods vary from direct tests, such as xenodiagnosis, which may include a combination of molecular procedures [21], to immunological techniques using different antigens (native or recombinant) [22, 23]. The high variability in sensitivity and specificity between methods has been related not only to the type of laboratory technique or antigen used, but also to geographical differences in parasite strains, as well as genetic differences between human populations, which may contribute to discrepancies in serologic tests [24]. This has led to a series of recommendations from the Pan American Health Organization (PAHO) and national guidelines [25–27], which recommend the use of two serologic tests in parallel for confirmatory purposes, with a sensitivity of at least 98% [28] to correctly diagnose the disease. However, when serologic tests are inconclusive, some guidelines recommend molecular tests such as PCR, which is the technique of choice for the confirmatory diagnosis of *T*. *cruzi* parasitemia [29]. The WHO recommends PCR as a confirmatory test after blood donor selection, in the diagnosis of acute or congenital infection, or for therapeutic follow-up after diagnosis of acute infection [28]. However, conventional PCR is not suitable for the diagnosis of chronic CD due to the low parasitemia in the chronic phase, which, together with the different extraction systems, blood sample preservation and DNA purification, makes PCR sensitivity around 50–90%, while its specificity remains close to 100% [30]. Due to the above-mentioned drawbacks, and despite its high specificity, PCR is only recommended when serologic tests are inconclusive, according to the recommendations of expert committees in different countries, such as Brazil, Chile, USA or Spain [25–27].

To address the challenges posed by patients with low parasitemia, such as the chronically ill or those requiring evaluation of treatment efficacy [31–33], new molecular methods have been developed over the last two decades. These methods aim to overcome the problems inherent in the disease itself or in the purification of DNA from blood samples. Thus, the use of chaotropic agents such as guanidine hydrochloride, which denature the proteins in the blood sample prior to DNA purification, new DNA extraction systems [17] and the use of specific primers that increase sensitivity [34, 35], especially for those hospital centers where the extraction of nucleic acids is carried out with automatic equipment and in which the extraction efficiency is not the most effective, this together with the development of PCR methodologies to increase sensitivity such as quantitative PCR (qPCR) [18] capable of detecting low concentrations of DNA (0.01 parasites in the sample), have gained ground. Also new amplification systems such as isothermal amplification (LAMP) [17, 36] that facilitate the implementation of molecular diagnosis are used. All of them aim to facilitate and increase the detection of the parasite in patients.

As in other diseases such as visceral Leishmaniasis [19, 20], in Chagas disease, the amount of circulating DNA of parasitic origin may be below the detection limits of molecular techniques. Therefore, new methods are sought to detect and demonstrate the active presence of the parasite. Among these methods is the detection in biological fluids of products of the particulate secretome, especially exovesicles of the parasite, where both proteins and nucleic acids make it possible to demonstrate the presence of metabolically active forms of the parasite [37].

Extracellular vesicles (EVs) are small membrane-coated vesicles released into the extracellular environment by almost any type of cell. EVs can be classified according to their size, biogenesis and composition; This classification includes: a) exosomes (20–100 nm), b) ectosomes (100–1000 nm) and c) apoptotic bodies (>1000 nm), among others [38, 39]. The composition of the EVs is complex and contains proteins, lipids, nucleic acids (DNA and RNA) [40, 41], including the EVs from *T*. *cruzi* [42]. EVs secretion by *T*. *cruzi* was first demonstrated by da Silveira et al., in 1979 [43] and, since then, several research groups have investigated the role of EVs in the pathogenesis of Chagas disease, demonstrating significant effects mainly on cell-cell communication, cell infection and evasion of the immune response [44, 45].

Although there are different methodologies both in the literature and commercially available, based on filtration systems, or through different chromatographic methods that facilitate the purification of the different exovesicles existing in biological fluids [41, 46–49] in the present work and as a proof of concept for the use of EVs in the molecular diagnosis of CD, we have followed the methodology considered the gold standard for the purification of exosomes, based on a mixed system of centrifugation/filtration/ultracentrifugation and the subsequent verification of the EVs purified by electron microscopy and nanoparticle tracking analysis (NTA), and which has been previously described by our work group [44, 50].

The presence of EVs of *T*. *cruzi* in the serum was previously demonstrated, and specifically, the presence of these EVs forming immunocomplexes containing specific *T*. *cruzi* proteins and without orthologues in other species in the serum of chronic patients with CD [50].

As a proof of concept, this paper demonstrates how the use of the parasite’s cell-free DNA (cfDNA) as well as the EVs of the parasite’s secretome present in the serum of chronic patients as “containers” that transport parasite DNA, can be used for molecular diagnosis of CD.

## Material and methods

### Human blood samples

Both, the blood and serum samples (n = 448) used in this study come from patients with symptoms compatible with CD or clinical suspicion and who attended the Hospital Universitario y Politécnico La Fe (HUyP-La Fe), Valencia, Spain during the years 2011–2020 where they were serologically analyzed with the LiaisonXL murex chemiluminescence kit and titrated with the Trinity Biotech IFA kit. Immunologically positive samples have been used in this study.

The samples included patients with suspected disease or with overt symptoms. These samples can be considered representative of the different population groups affected by CD, mostly adults (n 313), mostly born in Bolivia, with the exception of children born in Spain to mothers with Chagas disease (n 135). Some corresponded to patients who had received treatment for CD and who came for re-evaluation as follow-up, and patients with compromised immunity with post-transplant immunosuppression therapy; moreover, one of the patients was HIV seropositive. According to sex, 302 (67.4%) patients were female and 146 (32.6%) were male. All of the patients gave written informed consent before starting the study and all protocols were approved on March 4, 2011. The study was expanded to include the use to isolate and purify EVs from circulating serum and cell-free DNA (cfDNA) on September 26, 2018, with registration numbers 201102400000408 and 672/CEIH/2018 respectively, by the ethics committee of Granada University.

### DNA isolation method from peripheral blood

Peripheral blood samples (5 mL) were collected in Vacutainer vacuum blood tubes with EDTA and then subjected to a specific lysis pretreatment before proceeding to the DNA isolation methods. Pretreatment consisted of mixing 1:1 blood and denaturing lysis buffer (6M guanidine hydrochloride, 10 mM urea, 10 mM TRIS-HCL, 20% (v/v) Triton X-100; pH was adjusted to 4.4) through brief shaking. The samples were incubated at 70°C for 10 min. Before proceeding to the next steps, the samples were kept for at least 48 h at room temperature.

The automated purification protocol of the Maxwell Blood DNA Purification kit (Promega Biotech) was chosen according to the guidelines approved by HUyP-La Fe and was used following the manufacturer’s instructions. The initial volume for each of the blood sample was 400 μL of pretreated peripheral blood.

### Isolation of circulating cell-free parasite DNA (cfDNA) from serum

For the serum samples, 5 mL of blood were taken in the BD Vacutainer SST II Advance tubes (Reference. 366468). Once vortexed for 5 min, each tube was centrifuged at 1,500 x g for 10 min and approximately 2.0 mL of serum were collected to perform the different tests.

The samples used for the assay of this protocol were 4 serum pools and two individual serum samples from patients with digestive disorders. Two pools were formed by three patients with cardiac pathology each, and two pools formed by three patients with non-specific symptoms (S1 Table).

For the isolation of circulating DNA (cfDNA) of the parasite from the patient’s serum, the MagMAXTM cell-free DNA isolation kit (Thermo Fisher Scientific) was used and the manufacturer’s instructions were followed. First, the 100 μL serum pools were centrifuged at 1,600 x g 10 min at 4°C. Once the first centrifugation was completed, the supernatant was subjected to a second centrifugation at the same speed and for the same time as the previous one in order to remove any cellular debris in the serum. Next, the proteins contained in the supernatant were digested for 20 min at 60°C with 2 μL of Proteinase k (20 μg/mL). To the digestion product, 150 μL of buffer (30mTris-HCl pH 8.0, 10mM EDTA, 1% SDS) were added together with 5 μL of magnetic bead solution. The resulting volume (255 μL) of the mixture was vortexed intensively for 10 min and then centrifuged at 14,000 x g for 10 s. The resulting pellet was washed twice, first with 500 μL of wash buffer and then with 500 μL of 80% ethanol, and subjected to centrifugation (20 s at 1,3000 x g). Finally, the pellet was dried and resuspended in 50 μL Milli-Q water.

### DNA isolation from serum exovesicles (EVs-DNA)

To improve the detection of parasite DNA in *T*. *cruzi* infected patients, DNA was isolated from exovesicles (EVs) present in serum. For this assay, 25 nested PCR negative and 25 nested PCR positive samples were selected as described below.

Purification of circulating EVs from serum was performed following the methods previously described by Díaz et al., 2017 [50] and Retana et al., 2019 [44], by a mixed ultracentrifugation-ultrafiltration procedure. For this purpose, 1 mL serum samples were each diluted (1:1) with PBS previously ultrafiltered through 0.22 μm pore filters. The diluted samples were first centrifuged at 3,500 x g for 10 min (4°C) to eliminate contamination by cells or cellular debris from the serum. The pellet obtained was discarded and the supernatant was ultrafiltered through sterile 0.45 μm pore filters (Millipore, USA) in order to remove apoptotic debris and particles remaining in the supernatant from the first centrifugation. The supernatant was subsequently ultracentrifuged in microtubes (Hitachi No 1508) at 110,000 × g for 2 h at 4°C in a Sorwal WX80 centrifuge with fixed-angle rotor (Fiberlite™ F50L-24 × 1.5). The resulting pellet was washed three times by ultracentrifugation in sterile PBS as described above and evaluated by transmission electron microscopy and NTA nanoparticle tracking analysis, as described in a previous paper [42].

For the isolation of DNA from EVs-DNA, the method described by Orrego et al., in 2020 [51] was followed. To do so, the sediment containing EVs from serum was treated with a total of 2 units of DNAase I at 37°C for 30 min to remove external DNA from the EVs. After treatment, the enzyme was inactivated with heat at 70°C in a solution containing 50 mM EDTA. After removal of free nucleic acids from the EVs suspension, 100 μL of EVs were lysed with 200 μL of lysis buffer (30 mMTris-HCl pH 8.0, 10 mM EDTA, 1% SDS) supplemented with 20 μL of proteinase K (0.1 mg/mL), shaken by vigorous pipetting and incubated at 56°C for 1 h. Finally, nucleic acid purification and precipitation were performed following the traditional phenol-chloroform method by mixing 320 μL of lysed EVs suspension with 320 μL of phenol-chloroform-isoamyl reagent, 25:24:1 (v/v) (Sigma). The mixture was shaken briefly and centrifuged at 14,000 x g for 10 min. The aqueous phase was then collected and the DNA was precipitated with 1/10 vol of 3M sodium acetate pH 5 and 2.5 vol of HPLC grade absolute ethanol. The mixture was incubated overnight at -20°C and centrifuged at 15,000 x g for 10 min at 4°C, the supernatant was discarded and the precipitate was washed twice with cold 70% ethanol. Finally, the pellet was dried in a Jouan Thermo speed Vac (RC1010) at 20°C and diluted in 20 μl Milli-Q water.

### Transmission electron microscopy

To corroborate by electron microscopy, the purification of EVs from serum, the ultracentrifuged pellet resulting from the purification process described above was fixed for 2h at room temperature in 2.5% glutaraldehyde in cacodylate buffer pH 7.2 and then resuspended in PBS for sample processing by negative staining. For this purpose, approximately 15 μL of the suspension were deposited on nickel 300 mesh grids, coated with a charcoal layer, for 10 min. The grids were then washed twice with ultrapure water for 1 min. The grids were negatively stained with 1% uranyl acetate for 1 min. After staining, the samples were dried on filter paper and observed under a Zeiss Libra 120 Plus transmission electron microscope at 120KV at the Centro de Instrumentación Científica (University of Granada facilities).

### Detection of *T*. *cruzi* DNA in biological samples by different PCR strategies

#### Genes used in the study

In this work, two specific, conserved and highly repeated genes in the *T*. *cruzi* genome, widely used in the diagnosis of Chagas disease, were analyzed: the kinetoplast DNA (kDNA) minirepeat regions, which represent the major sequence component of kDNA with about 120,000 copies per parasite [52, 53], and the nuclear satellite (SAT DNA) with 10^4^ to 10^5^ copies in the highly conserved parasite genome [34].

### PCR strategies

#### kDNA minirepeat regions and nested PCR

DNA from the 448 serologically positive samples, extracted using the Maxwell Blood DNA Purification Kit automated protocol (Promega Biotech), and the minirepeated region of the kDNA was amplified. The reaction mixture was made up of Go Taq1flexi 1X amplification buffer (25 mM dNTPs, 2.5 mM MgCl_2_, 1.0 U of Go Taq1(Promega, USA), 10 pmol of specific primers 121F and 122R (Table 1) and 10 μL of template. The final volume was 50 μL according to the methodology described previously [54]. Amplification conditions consisted of two cycles, each of 1 min at 98˚C and 2 min at 64˚C, followed by 33 cycles of 1 min at 94˚C, and 1 min at 64˚C, before a final extension at 72˚C for 10 min. In all cases, a DNA sample of *T*. *cruzi* from the culture as a positive control and a non-template control were used.

**Table 1:** Sequences of primers used in this study.

Name	5‘→3‘ Sequence	Tm (°C)	Type	Type (5‘→3‘)	Product length (bp)	Gene
SATF	GCAGTCGGCKGATCGTTTTCG	60.1	PCR	F	120	Nuclear satellite DNA
SATR	TTCAGRGTTGTTTGGTGTCCAGTG	58.5	PCR	R		
121F	AAATAATGTACCGGKGAGATGCATGA	65	PCR	F	330	kinetoplastid DNA
122R	GGTTCGATTGGGGTTGGTGTAATATA	66	PCR	R		
T3	TC TTT GGT GTG ATC GTT AC	58	nested PCR	F	150	kinetoplastid DNA
T4	TAC ATT CTA TTT CTT CTC TG	52	nested PCR	R		
Cruzi1	ASTCGGCTGATCGTTTTCGA	56.6	Real time qPCR	F		Nuclear satellite DNA
Cruzi2	AATTCCTCCAAGCAGCGGATA	56.4	Real time qPCR	R		
Cruzi3	Fam-CACACACTGGACACCAA-NFQ-MGB	52.2	Real time qPCR	Probe		

In all instances, the amplification result, although not visible on DNA electrophoresis, was subjected to nested PCR amplification. To this end, 2 μL of the PCR product were used as template, 0.2 μM concentration of T3 and T4 primers (Table 1), 0.2 mM of each dNTP, 3 mM MgCL_2_, 0.5 μL of GoTaq Flexi DNA Polymerase; the final volume was 50 μL. The nested PCR conditions were (1) 94°C 5 min, (2) (94°C 1 min, 48°C 1 min, 72°C 1 min) × 35, (3) 72°C 7 min, (4) 12°C indefinite.

Amplifications were performed on a GeneAmp PCR System 9700 thermal cycler, AB Applied Biosystems and the nested PCR amplification product was sequenced on a GenomeLab GeXP system. The results obtained from sequencing were aligned using BLAST programs and the identity and coverage of these sequences were studied.

### EVs-DNA and cfDNA PCR

Both, the EVs-DNA present in the serum and the cfDNA were amplified indistinctly with the primer pair 121F-122R corresponding to the kDNA and with the SATF and SATR primers for the nuclear satellite regions (Table 1) using for these amplifications 0, 5 μM of the primers, 0.2 mM of each dNTP, 2.5 mM MgCl_2,_ 0.005 μL DMSO, 0.125 μL GoTaq Flexi DNA Polymerase enzyme and 2 μL as DNA template from DNA purification, the final reaction volume was 25 μL. For the SAT primers, the protocol was (1) 95°C 5 min, (2) (94°C 10 sec, 65°C 10 sec, 72°C 10 sec) × 40, (3) 72°C 5 min, (4). Samples were kept at 12°C.

The final products were visualized on 2% agarose gel stained with Safe Sybr and developed on a ChemiDoc MP Imaging System (Biorad). Electrophoresis was performed at 100 mV for 30 min.

### Nuclear satellite DNA real-time quantitative PCR

To ascertain if it is possible to quantify the DNA present in the EVs, the nuclear satellite sequence was amplified using real-time quantitative PCR (qPCR) following the validated protocol by Ramírez et at 2015 [18] with some modifications. The standard curve was generated using a starting quantity of 1.7 ng/μl from *trypomastigote stage of the parasite* DNA. The detection limit for DNA was determined to be 1.7 fg, with an efficiency of 101% and an R^2 value of 0.99. Standard curve (S1 Fig) and DNA-EVs samples were amplified according to the manufacturer’s instructions for SsoAdvanced Universal Probes Supermix (BioRad 172–5281), using 700 nM of primers concentration and 200 nM of probe. The primers and probe sequences are shown in Table 1. DNA quantification was measured on light cycler Bio-Rad CFX96, cycling conditions (1) 95°C for 10 min, (2) (95°C for 15 sec, 58°C for 1 min) × 39 cycles. All assays were performed in triplicate, including a template-free negative control and *T*. *cruzi* DNA positive controls prepared in a freshly prepared dilution series.

## Results

### Amplification of DNA from peripheral blood

The sera of 448 immunologically positive subjects, as indicated above, came from patients who had visited the different medical services of the Hospital La Fe. The majority (313) of whom were born in American countries, mostly in Bolivia. While 135, namely the youngest, were born in Spain but of Latin American mothers.

**Fig 1:**
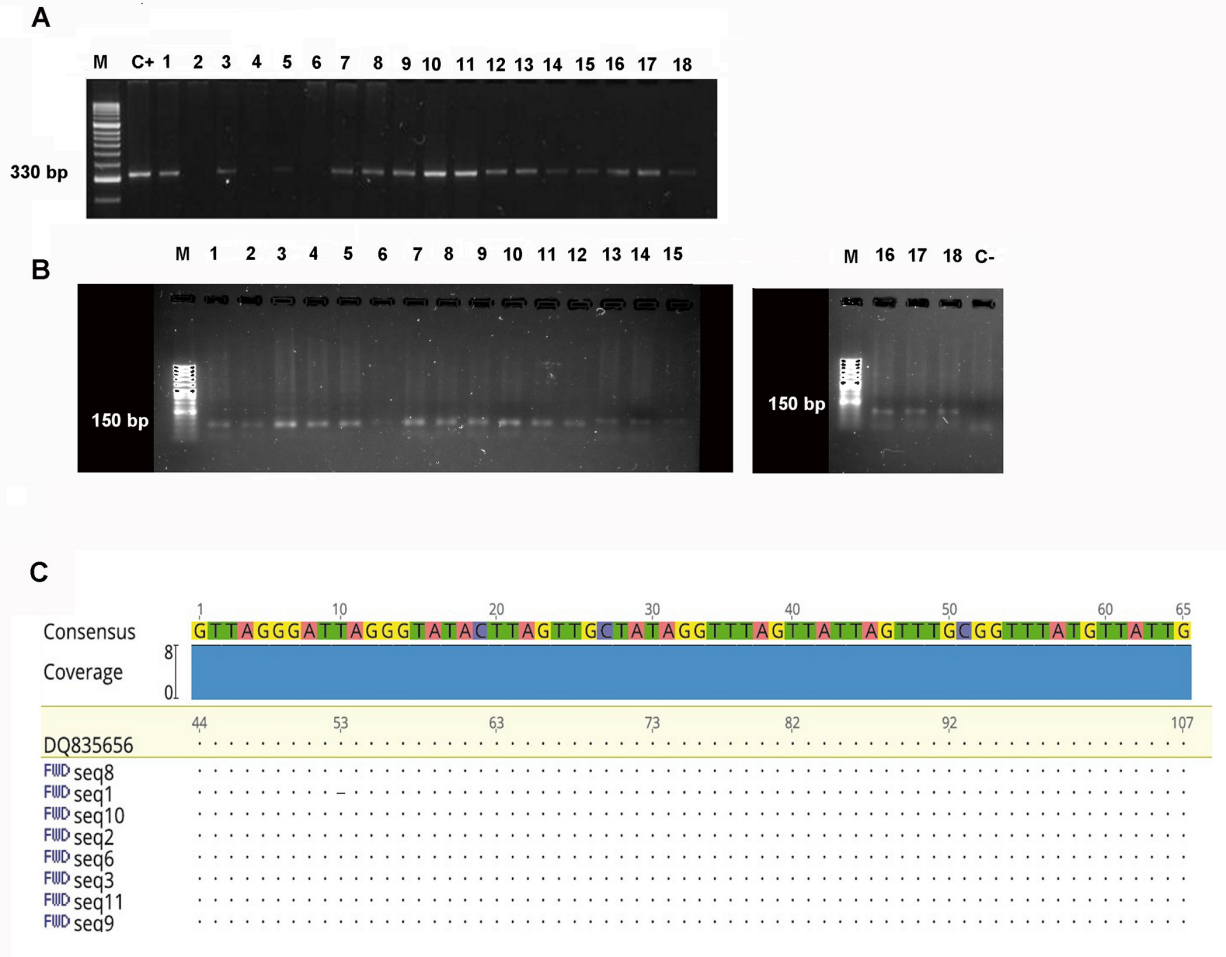
A: 2% agarose gel analysis of PCR-amplified products showing a 330 bp with 121 and 122 primers. PCR performed with peripheral blood DNA. Line 1, Hipper ladder II, Line 2, positive control, line 3 to 20 samples from 1 to 18 patients consecutively. B: 2% agarose gel showing a 150 bp band which belongs to nested PCR performed with T3 and T4 primers. C: Sequence alignment using the *T*. *cruzi* kDNA sequence with accession number DQ835656 as reference sequence. Our sequences were uploaded to GenBank with accession number as follow: OQ507819, OQ507820, OQ507821, OQ507822, OQ507823, OQ507824, OQ507825, OQ507826.

Fig 1A shows an example of 18 patients (15 positive and 3 negative) of the results of the first PCR with 330 bp amplicons. Nested PCR (corresponding to the kDNA) gave a positive result for 72 (16%). Eighteen samples are shown in which the 150 bp amplicon was visible in agarose electrophoresis (Fig 1B). Sequencing results of this amplicon are shown in Fig 1C. The results of the NCBI BLAST analysis of these sequences verified that the amplicon corresponded to a kDNA region of *T*. *cruzi*, 150 bp with coverage of 93% and an E of 2e-44 and an identity percentage of 97.4% (Fig 1C).

Analysis of the 72 samples, 448in which sample positivity was verified by nested PCR, resulted in 49 patients being female and 23 males. Mean age was 36.6 years (35 median) for females and 35.7 years (34 median) for males. Furthermore, positive patients were classified into 4 age ranges as shown in S2 Table. Thirty-four (5.6%) women were of childbearing age and 17 of whom were pregnant. Also, *T*. *cruzi* DNA was detected in the peripheral blood sample in the three children who were between 0 to 2 years old.

Most of the CD patients were born in Bolivia (n 70), one patient was from El Salvador and another one from Ecuador. S3 Table shows the distribution of origin in Bolivia, with Santa Cruz being the most represented region with 44.2% of the patients, followed by Sucre with 12.8%, Cochabamba with 10%, Potosí with 5.7%, La Paz (1.4%), Tarija (1.4%) and unidentified regions 25.2%.

S1 Table shows the conditions and age corresponding to the 72 positive nested PCR patients used in the study. Most of whom presented indeterminate symptoms (n 53), two had digestive disorders and nine patients suffered heart conditions. Of the total of 72 samples, 25 corresponded to patients who had not yet been treated with benznidazole. Also, it is noteworthy that of the 47 that had been treated only three remained PCR positive for *T*. *cruzi*. The period between the last dose of benznidazole and PCR was 1 year.

### DNA serum EVs and cfDNA amplification

#### Confirmation of circulating serum EVs

**Fig 2:**
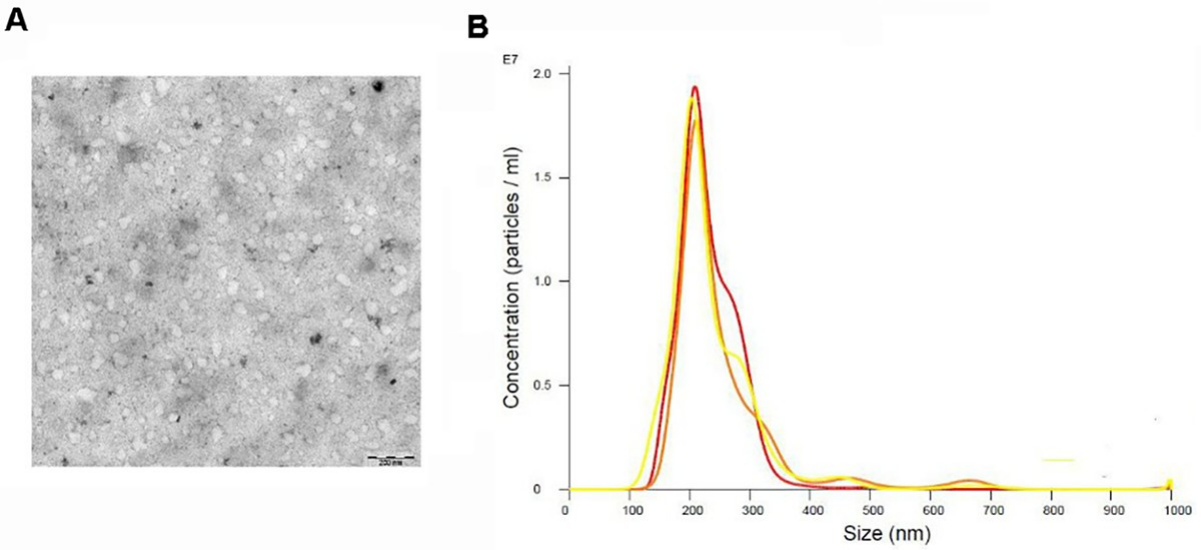
EVs characterization. A: isolated serum pool EVs observed under TEM. B; NTA showing the EVs populations. Isolation of extracellular vesicles by centrifugation filtration procedure from patients’ sera: A) Negative staining observed under Transmission Electron Microscopy (TEM) of the EVs obtained from the pellet after the purification of the EVs from the serum. The measurements of the EVs were carried out using the Imaje J software. (Scale bar: 200 nm). Mean diameter 119 nm; B) Nanoparticle tracking analysis and size distribution of EVs (the largest peak of number of particles corresponding to a size of 231nm).

TEM visualization of EVs from serums pool isolated by ultracentrifugation is shown in Fig 2A. The mean size of the EVs was 119.92 ±18.41 nm. NTA analysis (Fig 2B) revealed the different populations of these EVs in which the majority peak revealed a size of 163 nm. A second population less numerous of EVs with an average size of 231 nm was observed. Analysis of the total population of EVs showed a mode size of 208±84.8 nm; the mode being 163 nm. Also, few populations of larger size were observed which could be considered aggregates of the EVs purified from the circulating EVs in the serum of the patients studied.

#### Liquid biopsy

Samples were randomly selected using a stratified procedure between the two populations. Twenty-five of these were from the group of patients who did not test positive by nested PCR, and 25 sera were from the group of patients positive by nested PCR. Serum EV DNA (EVs-DNA) and circulating free cellular DNA (cfDNA) were purified from all the selected samples.

To perform PCR, as indicated in the Material and Methods section, primers 121F-122R and those corresponding to SAT satellite DNA were used with the DNA purified by these methods.

#### PCR analysis using DNA-EVs

**Fig 3:**
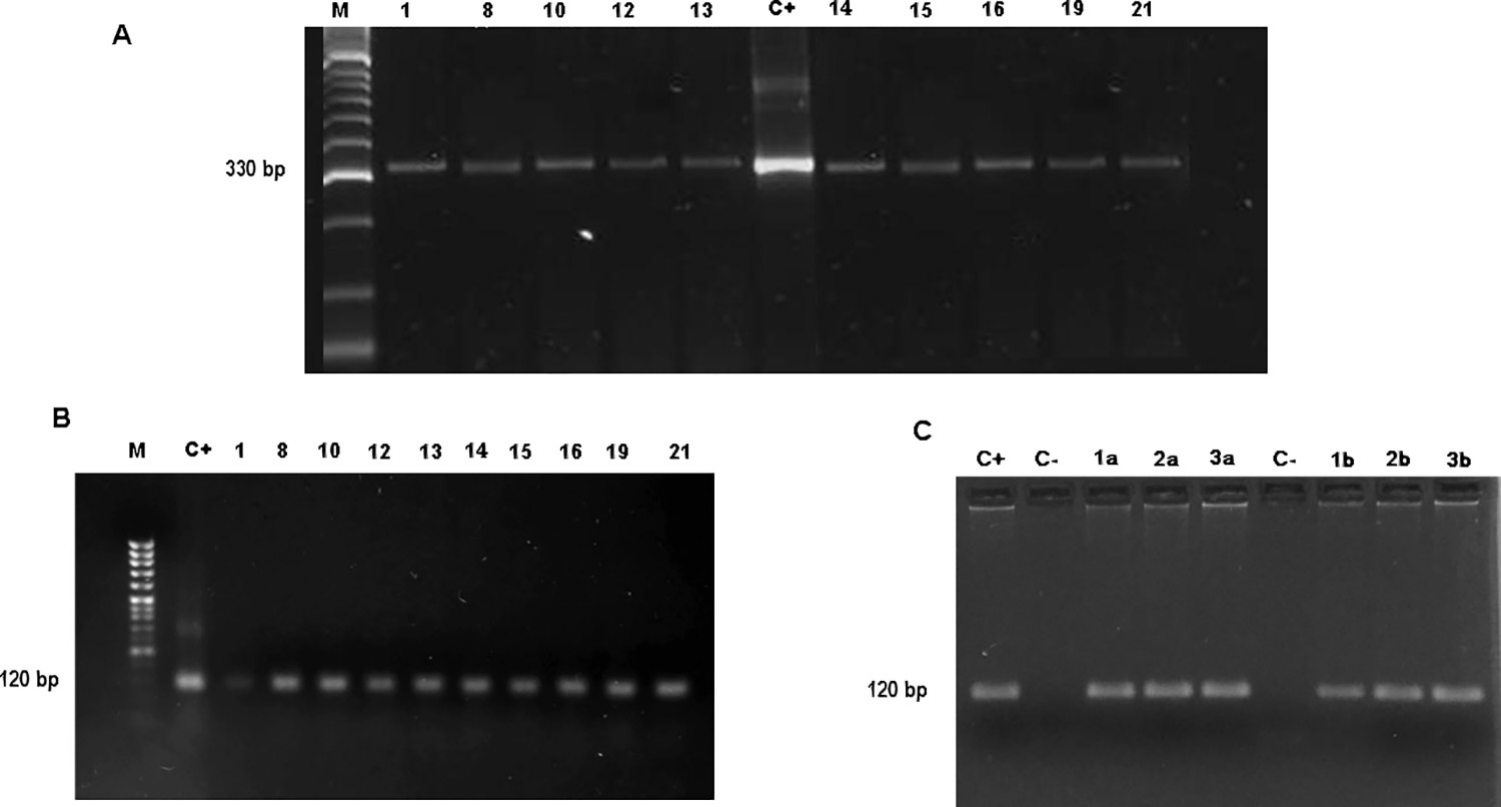
2% agarose gel electrophoresis analysis of PCR-amplified products. A: PCR performed with 121 and 122 primers and EVs-DNA. Line M, Hipper ladder II; Line 6, C+, positive control; line 2 to 5 and lines 7 to 11 samples of patients. B: PCR performed with SAT primers and EVs-DNA. Line M, Marker, Line 2, positive control; line 3 to 10 samples 1, 8, 10, 12, 13, 14, 15, 16, 19, 21, respectively. C: PCR performed with SAT primers and cfDNA. Line C+, positive control; Line C- non-template control; Line 3 to 5 samples 1a, 2a, 3a; line 6 non-template control; line 7 to 9 samples 1b, 2b, 3b.

The results of parasite EVs-DNA circulating in serum showed that twenty-five patients were positive for primers 121F-122R (Fig 3A) and all but one (24) were positive for SAT primers (Fig 3B).

#### PCR analysis using cfDNA

Isolated cfDNA was amplified by PCR with SAT primers. Fig 3C shows six positive samples (S1 Table) corresponding to patients with cardiac, digestive and indeterminate conditions with CD. The same samples were negative when amplified by PCR with primers 121F-122R.

#### EVs-DNA quantification

The Limit of detection (LOD) and limit of quantification (LOQ) of EVs-DNA (Table 2) were calculated following the methods described by Ramírez et al., 2015 [18]. In total, six EVs-DNA samples were analyzed using qPCR, and two of them were quantified to be 1.9 fg and 2.35 fg, respectively. The remaining samples yielded positive results but could not be interpolated on the standard curve.

**Table 2:** Analysis of results obtained for qPCR assay.

Patient number	Mean ct	Limit of detection (LOD) (fg)	Limit of quantification (LOQ) (1.7 fg)
8	31.5597125		1.9
10	32.19097398	1.22	
12	32.99075464	0.69	
13	31.23063642		2.35
14	32.83637534	0.9	
15	32.526172	0.95	

## Discussion

In the last decades, the distribution of CD has spread due to human migration for economic reasons from geographical areas of Central and South America, where hematophagous vector transmission is still the case, to non-endemic areas of North America, Europe, Japan or Australia. Spain, Italy and Switzerland are the three European countries [55] with highest levels of Latin American communities, especially Spain, related to their common language as well as historical and cultural links. The health authorities in Spain and Tuscany (Italy), areas with a high level of Latin American migrants have therefore adopted standards and recommendations on hemodonations [56], and transplant donors with regard to the detection of patients infected with *T*. *cruzi* in order to prevent human-to-human transmission of the disease [2, 57]. Similarly, regulations have been adopted for the screening of pregnant women originating from endemic countries and for which both the mother’s and newborn’s blood are tested [58, 59] in order to establish a treatment, which is well tolerated and effective in this type of patient. All this has prompted the standardization of diagnostic methods for CD in health centers and especially for chronically ill patients, in whom parasitemia levels are practically non-existent.

Classical parasitological diagnostic methods, such as visualization of trypomastigote forms in blood smears or thick drop, or even trypomastigote concentration techniques by centrifugation in microhematocrit tubes, have been used to diagnose CD [60, 61]. They show high specificity but very low sensitivity, especially in chronic patients. These methods may be applicable in diagnosis when the disease is developed with high parasitemia in the acute phase or after reactivation of circulating parasitemia in immunosuppressed chronic patients [60]. Only culture and xenodiagnosis can be used to directly detect *T*. *cruzi* infections in the chronic phase. These techniques are, however, not useful in clinical practice, especially in Europe, due to the limitations of insect vector handling and the delay in obtaining results, which limits their usefulness in diagnostic studies and in evaluating treatment efficacy [62, 63]. Moreover, the evaluation of different techniques shows that in the case of low parasitemia, PCR is at least 27 times more sensitive than blood culture [64]. Also, Ramirez et al., 2015 applied an qPCR and a LOQ was estimated at 1.53 par. eq./mL and 0.90 par. eq./mL for SatDNA and kDNA respectively[18, 34]. These results were similar to the amount of *T*. *cruzi* specific DNA measured in our work (a LOD of 1.9 fg and a LOQ of 2.35 fg) when nuclear satellite DNA was quantified in EVs.

The course of the disease is characterized by elevated IgM levels during the initial acute phase of the disease and the appearance of IgGs that persist throughout the disease [65]. Thus, immunological diagnostic techniques are the gold standard for the diagnosis of CD in chronic patients, even in asymptomatic cases. The limiting factors in serological studies are the possible cross-reactivity with *Leishmania* spp. or *Trypanosoma rangeli* infections in those geographical areas where the disease was acquired and these trypanosomatids coexist [62, 66]. Another drawback could be the lack of sensitivity to the different parasite strains that affect humans throughout the Americas. They may present antigenic variability, which requires, if possible, the use of antigens of different geographical origin and different nature [24, 67] according to the recommendations of the different committees on CD. Individuals are considered infected when they have a positive result in at least two serological techniques using different antigens. A third technique is required in case of discrepancy of indeterminate immunological results [25–27, 68], or the use of a mixture of antigens that minimize discrepancies [22, 69]. The other significant case that invalidates immunological techniques is the diagnosis in newborns from infected mothers due to the presence of IgGs from the mother to the fetus by transplacental transfer [64, 70–72].

In cases that require the detection of the direct presence of the parasite in blood, when doubtful or inconsistent serological results are obtained and when the efficacy of the patient’s treatment is evaluated, PCR methods capable of detecting the presence of parasite nucleic acids in blood [73] are needed to determine the unequivocal presence of the parasite. However, this technique, has a number of limitations that can alter the results and that must be considered [74, 75] such us the volume of blood processed, the correct handling of the blood (use of the chaotropic agent guanidine, time and treatment of the lysis process), the manual or automatic DNA extraction procedures, the presence and especially the elimination of PCR inhibitors, the primers used, etc. For all of these reasons, the quest for new procedures and methodologies in the extraction of nucleic acids to be applied to minimize false negative results continues.

The samples studied, reflect the Latin American population living and working in the area of influence of the Hospital La Fe in Valencia. Data from INE (National Institute of Statistics) for the year 2021 show that 17,847 people born in Bolivia reside in the Valencian Community, 39.1% are male and 60.9% are female.

In order to be able to select among the serologically positive patients those who could have parasitemia in the blood, and after carrying out the diagnostic PCR with the primer 121F-122R used in the hospital, and to increase the sensitivity of the detection, a nested PCR was performed on all the samples, in which the primers T3 and T4 designed for this purpose were used and which would amplify a band of 150 bp from the internal zone of the amplicon produced in the first PCR. The use of nested PCR using other genes with the products of the first PCR was already studied by Pereira et al., 2016 [76] and Ribeiro et al., 1999 [77], who concluded that the sensitivity of a series of 3 nested PCRs using primers specific for conserved regions of the Kinetoplast is able to detect 1 parasite per ml of blood, increasing the sensitivity of the PCR by about 10,000 times. Other authors such as Marcon et al., 2002 [50], or Riera et al., 2006 [78] employed nested PCR after conventional PCR using TCZ1 and TCZ2 and internal primers TCZ3 and TCZ4 to evidence parasitization in a neonate. Also, Pereira et al., 2016 [76] used nested PCR for the products of a PCR using the TCZ1 and TCZ2 primers previously mentioned to detect CD positivity in a population of elderly people suffering from cardiac problems and in whom the involvement of *T*. *cruzi* in these pathologies was not suspected.

Sixteen percent of the total 448 immunologically positive patients tested positive in nested PCR.

The amplicons obtained were visualized by electrophoresis resulting in a band of 150 bp. Sequencing of the amplicons showed that they correspond to an internal region of the *T*. *cruzi* kDNA described by Burgos et al., 2009 [79], with a coverage of 93% and a percentage of identity of 97.4%, Fig 1.

The treatment of choice usually prescribed for CD-positive patients in Spain consists of 5 mg/kg benznidazole (BZ; N-benzyl-2-nitroimidazole acetamide) daily for 60 days. It is noteworthy that, of the 47 selected patients who had been treated, three tested positive post-treatment in nested PCR for *T*. *cruzi*. It was not ruled out that the treatment was not completed as a consequence of the side effects involved and the consequent interruption by the patient, and in one case the patient stated that he had returned to his country, not ruling out reinfection.

We also evaluated whether cfDNA present in the serum of CCD patients could be used as a template for specific PCRs to improve diagnosis in CCD and in neonates where parasitemia is very low and almost undetectable by other diagnostic procedures. The results of the analysis were 100% positive. This confirms that serum is a good sample to carry out DNA extraction to be used in the PCR detection of *T*. *cruzi*, which was already determined by Russomando et al., 1992 [80] and in the review carried out by Weerakoon et al., 2016 [81] and others [82, 83] where the use of cfDNA in different biological samples in the diagnosis of different parasitosis was encouraged.

In our study, patients who tested positive using EVs-DNA or cfDNA as PCR templates and patients who tested negative had similar characteristics; all of them had CCD. However, it is important to note that there was a higher proportion of positive patients who received no or incomplete treatment (7 of 25) compared with negative patients (4 of 25) (see S4 Table). Taking into account patient status, PCR was more successful in patients with known pathology compared to patients diagnosed as indeterminate (11 and 14 respectively for PCR positive vs. 4 and 18 for PCR negative). Of note, age and sex did not show significance within this population.

The results obtained in our cfDNA (nuclear DNA or mitochondrial KDNA) of parasitic origin would come from circulating parasites coming from either lysis or apoptosis of free or intracellular parasitic forms, or transported in elements secreted by the parasitic forms. With these sera we proceeded to the purification of EVs circulating in the serum, and the purification of the EVs from the sera of the patients was evidenced by both, electron microscopy and NTA. The term EVs designates the set of various EVs released by cells and delimited by a lipid bilayer that are released into the extracellular space by prokaryotic and eukaryotic cells [84].

EVs constitute cellular mechanisms of cell-to-cell communication over short or long distances between cells acting as endocrine, paracrine, juxtacrine or autocrine signaling [44], through uptake of EVs or receptor-mediated interactions. Evidently, blood is the mechanism of transport and diffusion of these EVs. EVs are classified by their size, biogenesis and composition, exosomes with sizes between 20 to 200 nm, ectosomes between 200 to 1000 nm and apoptotic vesicles of a larger size [41]. Exosomes contain functional molecules (including proteins, nucleic acids and lipids) derived from their cells of origin, being present in all biological fluids where they have been sought, urine and saliva and undoubtedly in blood [85–87]. Due to the protection of the lipid bilayer, exosomes are relatively stable and proteins and other molecules transported in these small vesicles are protected from degradation. Their composition is quite complex, including a wide variety of lipids, proteins, different populations of RNAs, ssDNA, and metabolites [42]. Typically, the internal volume of an exosome ranges from 20 to 90 nm^3^, suggesting that a prototypical exosome would contain approximately 100 proteins and 10,000 nucleotides [88].

In the present study, we have demonstrated the presence of parasite DNA, both from the kinetoplast and the nucleus. Furthermore, we have shown that it is possible to use it for diagnosis due to the high number of positively diagnosed patients, similar to what is found in experiments using total patient blood. The presence of parasite EVs in the serum of patients with CD was already described by some of us in 2017 [50] and these EVs appeared circulating in the serum forming immunocomplexes in patients with different Chagas pathologies, which denoted the presence of metabolically active forms of the parasite and capable of actively releasing these EVs. The fact that EVs capable of amplifying nuclear DNA and amplifying kDNA are found in the serum of those affected by CCD highlights the origin of circulating EVs, some originating from the endocytic pathway after fusion of late endosomes/mulvesicular bodies (MVB) of the parasite and the others of mitochondrial origin of the pathogen such as those recently described in the serum [89] and known as Mitovesicles, and involved in inflammatory processes [90].

It is the first time that this type of EVs carrying kDNA of strict mitochondrial origin has been described in the serum of patients affected by CCD, indicating the active presence of the parasite in the patients in whom it is found and constituting a marker for the presence of an active infection.

EVs have been used as liquid biopsy in numerous assays for tumor diagnosis [91–93], however, the use of EVs-DNA for the diagnosis of pathogens and especially intracellular pathogens has hardly been addressed so far. Cho et al., 2020 [94] showed that PCR using exosome DNA from isolates of patients affected by tuberculosis shows higher sensitivity than conventional PCR diagnosis using total DNA for PCR. To our knowledge, this is the first study to report the detection of *T*. *cruzi* DNA from circulating EVs in the serum of patients using two sets of primers, i.e., those that detect kinetoplast DNA minicircles (121F-122R) and those that recognize nuclear DNA with the SATF and SATR primers.

From the results of the present work, it is evident that both cfDNA and EVs-DNA from the serum of CCD patients constitute useful and effective biological samples to demonstrate by PCR the active presence of the parasite, in clinical cases (neonates and studies of the efficacy of treatment) in which it is necessary to demonstrate the presence of the parasite.

## Supporting information

S1 Table Patients information. (XLSX)

S2 Table Patients age ranges. (XLSX)

S3 Table Distribution of origin in Bolivia in regions. (XLSX)

S4 Table Positive patients and characteristics. (XLSX)

S1 Fig qPCR standard curve and samples. (TIF)

S1 Raw images (PDF)
