# Supplementary figures and images for: Use of sera cell free DNA (cfDNA) and exovesicle-DNA for the molecular diagnosis of chronic Chagas disease
Source: PLoS One. 2023 Sep 8;18(9):e0282814. doi: 10.1371/journal.pone.0282814 (PMC10490946; doi:10.1371/journal.pone.0282814)

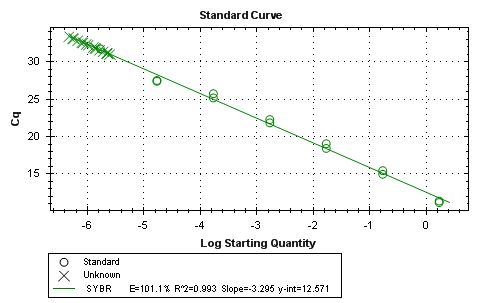

Supplement: S1 Fig — (TIF) [file pone.0282814.s005.tif]

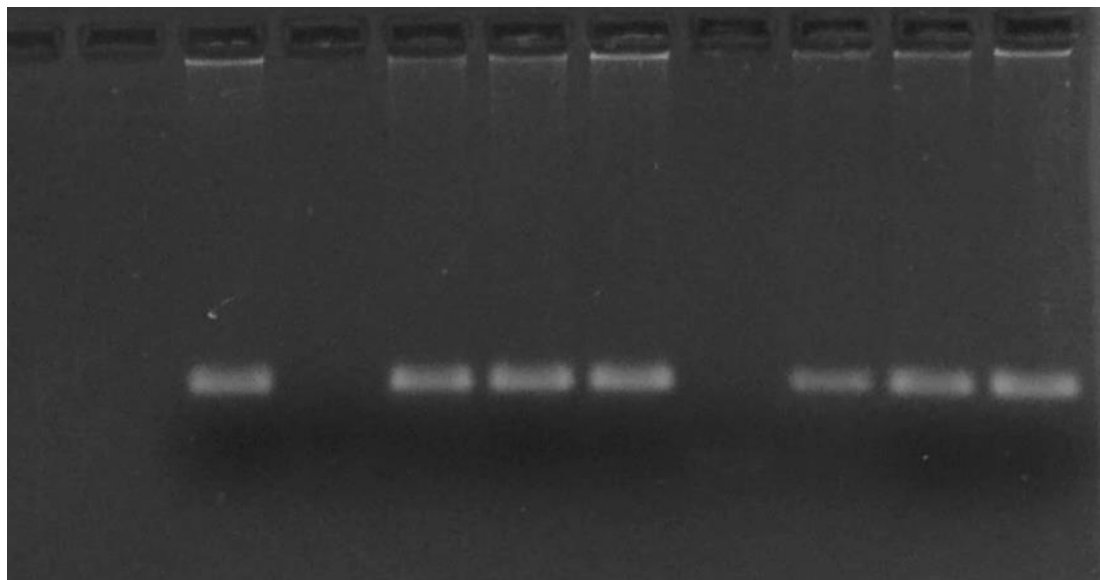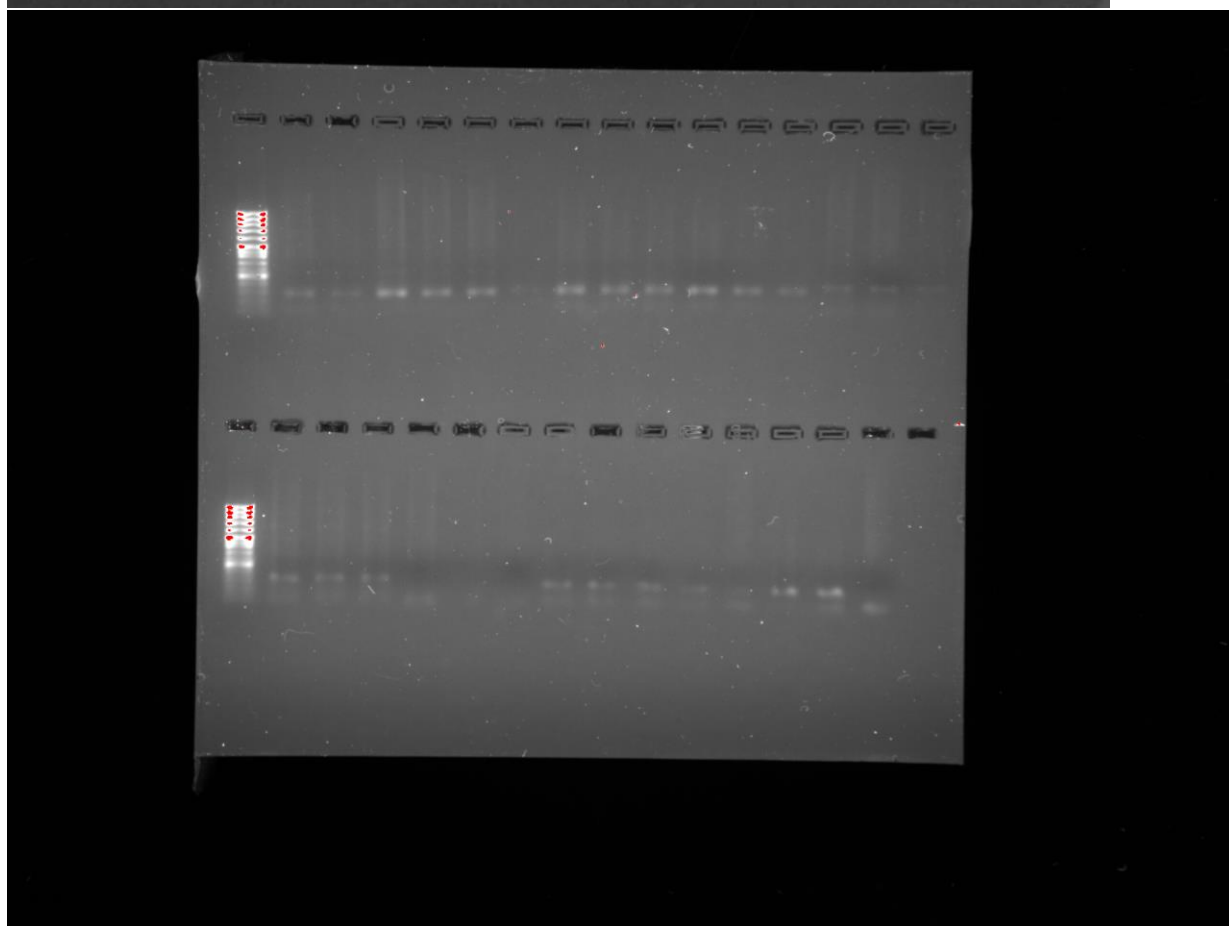

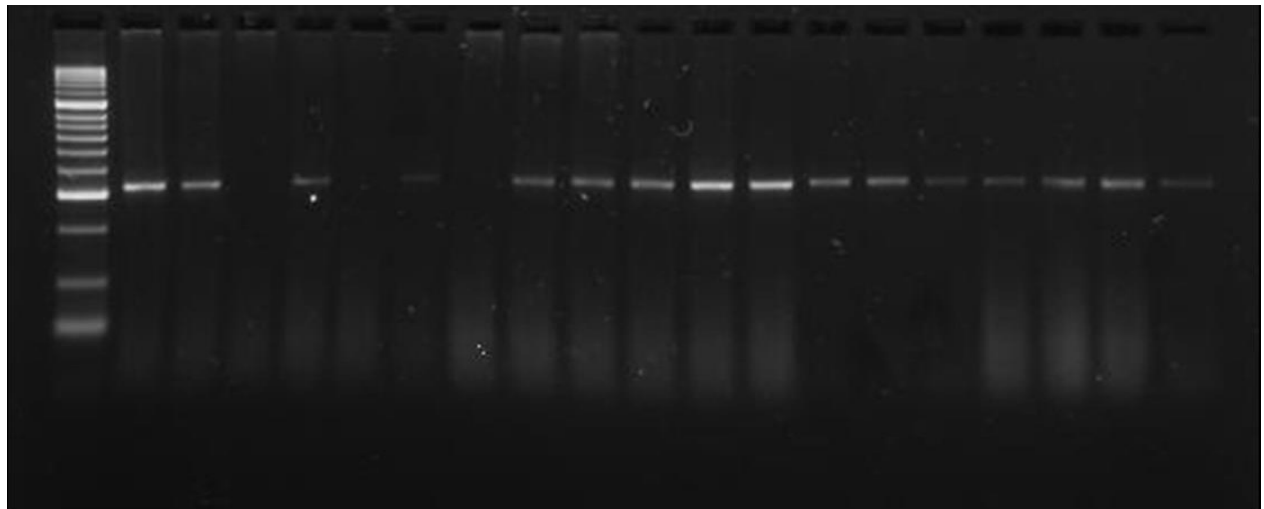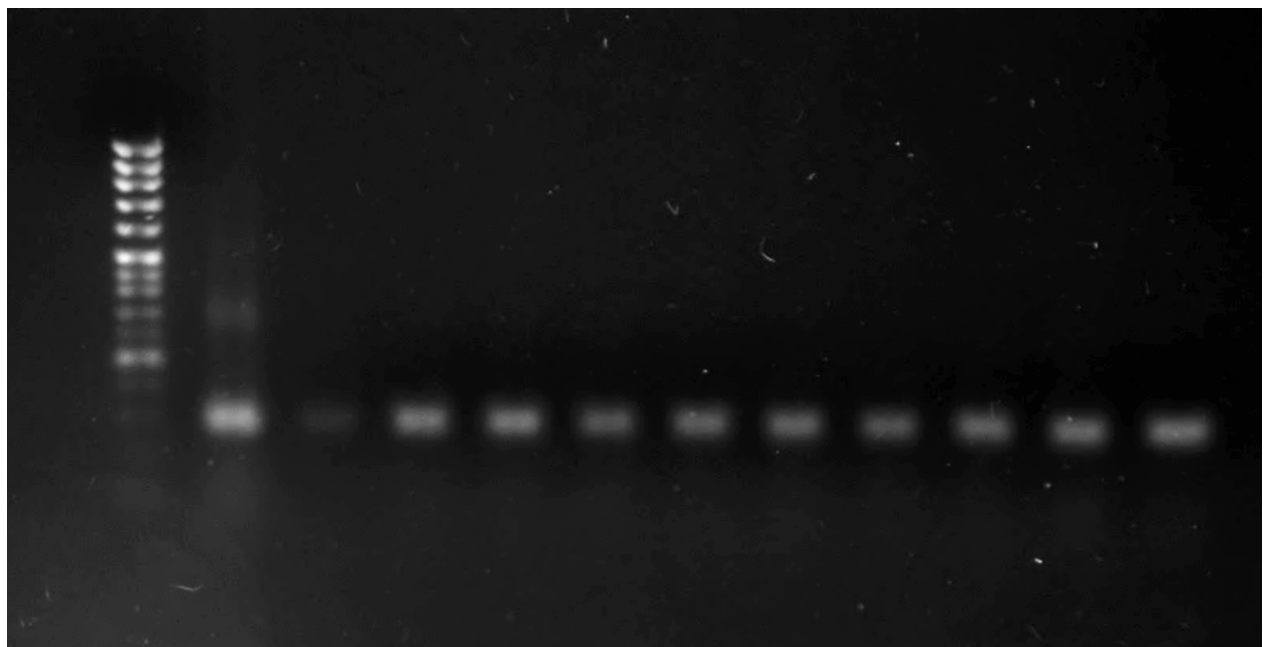

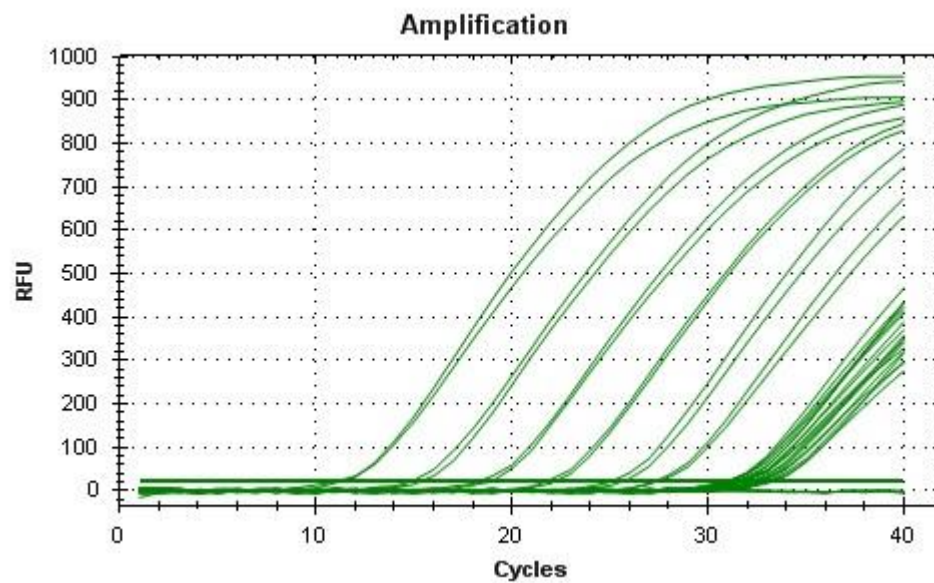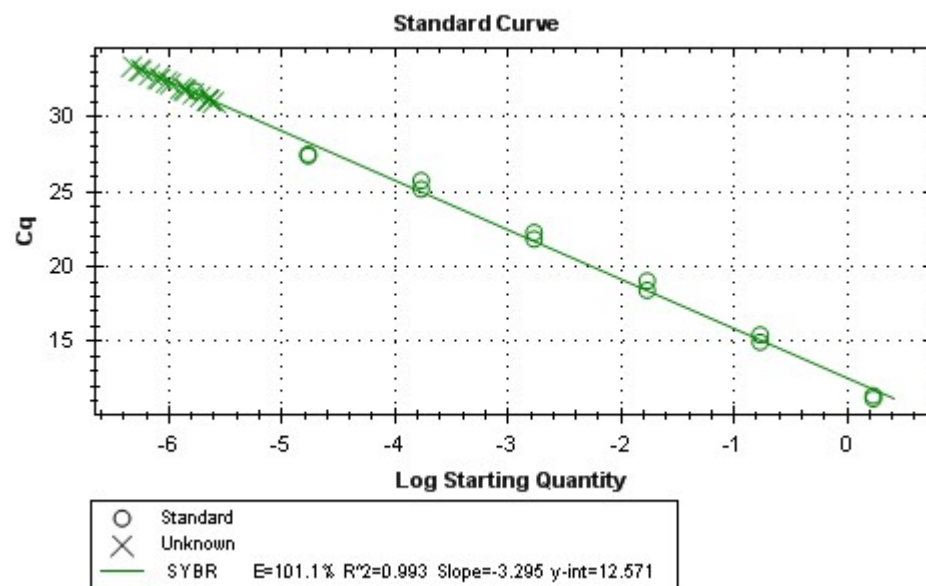

Supplement: S1 Raw images — (PDF) [file pone.0282814.s006.pdf]
